# Methylene blue in the treatment and prevention of ifosfamide-induced encephalopathy: report of 12 cases and a review of the literature

**DOI:** 10.1054/bjoc.1999.0917

**Published:** 2000-01-18

**Authors:** J Pelgrims, F De Vos, J Van den Brande, D Schrijvers, A Prové, J B Vermorken

**Affiliations:** Department of Medical Oncology, University Hospital Antwerp, Wilrijkstraat 10, Edegem, B-2650, Belgium

**Keywords:** ifosfamide, methylene blue, encephalopathy

## Abstract

Ifosfamide is an alkylating agent used in the treatment of a variety of solid tumours. Ten to 15% of patients treated with ifosfamide develop an encephalopathy. Methylene blue (MB) may be used in the treatment of this encephalopathy. The purpose of this study was to evaluate the neuroprotective effect of MB in these patients and to review the literature. Between 1993 and 1997, 52 patients (age 16–77 years) with solid tumours were treated with ifosfamide in dosages ranging from 3 to 5 g m^−2^q3w when given in combination schedules and up to 12 g m^−2^q4w when given as a single agent. Twelve patients developed central nervous system (CNS) depression, defined as National Cancer Institute Common Toxicity Criteria (NCI-CTC) neurocortical toxicity grade 2 or higher. Eight were treated with MB at a dose of 6 × 50 mg day^−1^intravenously (i.v.). Four recovered fully within 24 h, two recovered partially after 24 h and completely after 48 h while two recovered only after 72 h. Four patients did not receive MB and all recovered only after 48 h. Three patients received prophylaxis with MB at a dose of 4 × 50 mg day^−1^i.v. for the subsequent chemotherapy cycles. Two developed milder encephalopathy; one had no CNS depression at all. We conclude that MB is an effective treatment for ifosfamide-induced encephalopathy. Our findings suggest that it may also be used as a prophylactic agent. © 2000 Cancer Research Campaign


*British Journal of Cancer* (2000) **82**(2), 291–294
© 2000 Cancer Research Campaign
Article no. bjoc.1999.0917

### Methylene blue in the treatment and prevention of ifosfamide-induced encephalopathy: report of 12 cases and a review of the literature

**J Pelgrims, F De Vos, J Van den Brande, D Schrijvers, A Provè and JB Vermorken**

Department of Medical Oncology, University Hospital Antwerp, Wilrijkstraat 10, B-2650 Edegem, Belgium

**Summary** Ifosfamide is an alkylating agent used in the treatment of a variety of solid tumours. Ten to 15% of patients treated with ifosfamide develop an encephalopathy. Methylene blue (MB) may be used in the treatment of this encephalopathy. The purpose of this study was to evaluate the neuroprotective effect of MB in these patients and to review the literature. Between 1993 and 1997, 52 patients (age 16–77 years) with solid tumours were treated with ifosfamide in dosages ranging from 3 to 5 g $m^{-2}$ q3w when given in combination schedules and up to 12 g $m^{-2}$ q4w when given as a single agent. Twelve patients developed central nervous system (CNS) depression, defined as National Cancer Institute Common Toxicity Criteria (NCI-CTC) neurocortical toxicity grade 2 or higher. Eight were treated with MB at a dose of $6 \times 50$ mg $day^{-1}$ intravenously (i.v.). Four recovered fully within 24 h, two recovered partially after 24 h and completely after 48 h while two recovered only after 72 h. Four patients did not receive MB and all recovered only after 48 h. Three patients received prophylaxis with MB at a dose of $4 \times 50$ mg $day^{-1}$ i.v. for the subsequent chemotherapy cycles. Two developed milder encephalopathy; one had no CNS depression at all. We conclude that MB is an effective treatment for ifosfamide-induced encephalopathy. Our findings suggest that it may also be used as a prophylactic agent. © 2000 Cancer Research Campaign

**Keywords:** ifosfamide; methylene blue; encephalopathy

Ifosfamide is an alkylating agent used in the treatment of many solid tumours. Its use may be limited by specific side effects. One of the most serious of these is central nervous system (CNS) depression known as ifosfamide-induced encephalopathy. The clinical picture can range from mild somnolence or agitation over confusion or hallucinations to deep coma. Until recently, there was no effective treatment for this complication and when it occurred ifosfamide administration had to be discontinued. There have been some sporadic reports of patients with ifosfamide-induced encephalopathy being successfully treated with methylene blue (Küpfer et al, 1994; Zulian et al, 1995; Ferrero et al, 1995; Demandt and Wandt, 1996; Alonso et al, 1996). In this paper we present our experience with methylene blue (MB) in the treatment and prevention of ifosfamide-induced encephalopathy and give a review of the literature.

### PATIENTS AND METHODS

The files of all patients treated with ifosfamide- containing regimens between 1993 and 1997 were studied retrospectively. Ifosfamide was used both as single agent or in combination chemotherapy in doses ranging from 3 to 5 g $m^{-2}$ every 3 weeks when used in combination therapy and up to 12 g $m^{-2}$ every 4 weeks when used as single agent.

*Received 18 March 1999*
*Revised 16 July 1999*
*Accepted 2 August 1999*

*Correspondence to:* JB Vermorken

Ifosfamide was administered in different infusion schemes varying from fractionated, 1-h infusions on 3 consecutive days in the combination treatments to a continuous 72-h infusion in the high dose schedules. All patients received standard prehydration with normal saline. Haematological and non-haematological toxicities were classified according to the National Cancer Institute – Common Toxicity Criteria (NCI-CTC) (Van Tongelen, 1994). Encephalopathy grading is shown in Table 1. Neurocortical toxicity was graded as significant when it was at least grade 2.

At that time routine electroencephalogram was not performed for this indication at our department.

The time to recovery from the encephalopathy in the group of patients treated with methylene blue and in the group without treatment was recorded.

### RESULTS

In total, 52 patients were treated with ifosfamide. Most of these patients had sarcoma (32%), lung (17%) or cervical carcinoma (13%). Twelve patients (23%) developed NCI-CTC neurotoxicity grade 2 or more (Table 2). The infusion of ifosfamide was not stopped in any of these patients. When compared to the 40 patients without neurotoxicity, there was no difference in renal function (data not shown). There were fewer patients with pelvic localization of disease in the group who had neurotoxicity (16% vs 42%).

Eight patients were treated with MB in a dose of $6 \times 50$ mg $day^{-1}$ intravenously (i.v.), and four were not treated because the attending physician did not judge the situation, although grade 2 or more neurotoxicity, as severe. Methylene blue was started immediately upon clinical diagnosis.

**Table 1** NCI common toxicity criteria; neurocortical toxicity

| | Grade 0 | 1 | 2 | 3 | 4 |
|---|---|---|---|---|---|
| Symptoms | None | Mild somnolence or agitation | Moderate somnolence or agitation | Severe somnolence, agitation, confusion, disorientation or hallucinations | Coma, seizures, toxic psychosis |

**Table 2** Results

| Patient no. | Diagnosis | Toxicity grade | Dose ($g\ m^{-2}\ day^{-1}$) | Infusion time (h) | Methylene blue ($mg\ day^{-1}$ | Time of first symptom (day) | Time to recovery (h) | Prophylaxis ($mg\ day^{-1}$) | Second event |
|---|---|---|---|---|---|---|---|---|---|
| 1 | NSCLC | 2 | 1.5 $g\ m^{-2}$ days 1–3 | 1 | | 3 | 48 | | |
| 2 | Liposarcoma | 3 | 4 $g\ m^{-2}$ days 1–3 | 24 | | 5 | 48 | | |
| 3 | Ovarian carcinoma | 2 | 5 $g\ m^{-2}$ | 24 | | 1 | 48 | | |
| 4 | Chondrosarcoma | 2 | 4.7 $g\ m^{-2}$ days 1–3 | 24 | | 4 | 48 | | |
| 5 | Synovial sarcoma | 4 | 5 $g\ m^{-2}$ | 24 | 6 × 50 | 2 | 12 | | |
| 6 | Small-cell carcinoma | 3 | 4 $g\ m^{-2}$ | 24 | 6 × 50 | 8 | 12 | | |
| 7 | Stromal sarcoma | 3 | 3.2 $g\ m^{-2}$ days 1–3 | 24 | 6 × 50 | 4 | 12 | | |
| 8 | Neuro-endocrine tumour | 2 | 1.5 $g\ m^{-2}$ days 1–3 | 1 | 6 × 50 | 3 | 24 | | |
| 9 | Malignant schwannoma | 3 | 4 $g\ m^{-2}$ days 1–3 | 24 | 6 × 50 | 3 | 48 | 4 × 50 | grade 2 |
| 10 | Rhabdomyosarcoma | 3 | 2.5 $g\ m^{-2}$ day 1<br>3 $g\ m^{-2}$ day 2<br>3.5 $g\ m^{-2}$ day 3 | 12 | 6 × 50 | 2 | 48 | | |
| 11 | Angiosarcoma | 3 | 4 $g\ m^{-2}$ days 1–3 | 24 | 6 × 50 | 3 | 72 | 4 × 50 | grade 0 |
| 12 | Cystosarcoma phyloides | 3 | 4 $g\ m^{-2}$ days 1–3 | 24 | 6 × 50 | 3 | 72 | 4 × 50 | grade 2 |

Four of eight patients treated with MB recovered completely within the first 24 h, three of these within 12 h. One of them recovered within 1 h of a grade 4 neurotoxicity after the administration of MB.

Two patients had incomplete recovery after 24 h but recovered fully after 48 h. Two patients recovered after 72 h. The mental status of the patients without MB became normal after 48 h.

Three patients received prophylactic MB in a dose of 4 × 50 mg $day^{-1}$ i.v. for the next courses. Two of them developed an encephalopathy of lower grade and one had no subsequent evidence of neurotoxicity.

### DISCUSSION

Ifosfamide is a prodrug metabolized by cytochrome p450 to its active alkylating agents, 4-hydroxy-ifosfamide and isofosforamide mustard (Kaijser et al, 1994). Other non-alkylating metabolites are being formed, which may be responsible for the toxicity of ifosfamide (Kurowski et al, 1991).

The likelihood of ifosfamide-induced encephalopathy depends on the route of administration (Cerny et al, 1990). It is more common after oral than after i.v. administration. After i.v. administration, it occurs more frequently with a short infusion time. It is therefore generally accepted to give ifosfamide as a protracted or fractionated infusion.

The exact pathophysiological mechanisms responsible for the development of ifosfamide-induced encephalopathy are not known. Küpfer et al (1996) presented possible pathways by which ifosfamide metabolites can induce neurotoxicity. These hypotheses were based on the finding of glutaric acid and sarcosine in the urine of a patient with ifosfamide-induced encephalopathy. The same products are also found in the urine of patients with congenital glutaric aciduria. In these patients a metabolic dysfunction is caused by the absence of glutaryl CoA (type 1) or by a lack of electron transferring flavoproteins in the mitochondrial respiratory chain (type 2). Further investigations showed a relation between glutaric aciduria and chloroethylamine but not with any of the other metabolites of ifosfamide. This led to the conclusion that chloroethylamine may be the principal neurotoxic metabolite of ifosfamide.

Chloroethylamine conjugates with cystein, thus forming thialysine, which can be metabolized to thialysine ketimine. The latter can inhibit the electron-binding flavoproteins in the mitochondrial respiratory chain. Thialysine ketimine could have CNS effects on its own. The inhibition of the mitochondrial respiratory chain may also lead to a disturbance of the intracellular NAD/NADH balance with the accumulation of NADH. This in turn prevents the dehydrogenation of aldehydes, such as the ifosfamide metabolite chloracetaldehyde, which need NAD for their oxidation. Chloracetaldehyde is a potential neurotoxic substance. It is closely

© 2000 Cancer Research Campaign

**Table 3** Review of the literature

| Author (year) | Patients ($n$) | Ifosfamide dose (g $m^{-2}$ $day^{-1}$) | Methylene blue dose (mg $day^{-1}$) | Time to recovery (days) |
|---|---|---|---|---|
| Watkin (1989) | 18 | 5 | | 3 (1–12) |
| Merimsky (1992) | 2 | 5 | | fatal |
| | 2 | 1.8–2 × 4 | | 3–7 |
| | 1 | 1 × 5 | | 3–7 |
| Curtin (1991) | 6 | 2.5–5 | | 4 (2–13) |
| DiMaggio (1994) | 6 | 2.85–3.3 × 6 | | 4 (3–7) |
| Küpfer (1994) | 1 | 2.4 × 6 | 3 × 50 | 30 min |
| Zulian (1995) | 1 | 5 | 1 × 50 | 10 min |
| Ferrero (1995) | 1 | 2 × 3 | 100 | 1 |
| Demandt (1996) | 1 | 1.5 × 5 | 2 × 50 | 1 |
| Alonso (1996) | 1 | 2dl + 1.5 dl–2 | 1 × 60 | 5 hours (partial) |
| Koschuth (1996) | 1 | 1.5 × 5 | 2 × 50 | 8 |

related to chloralhydrate, a known hypnotic, and to acetaldehyde which is the neurotoxic metabolite of ethanol (Pratt et al, 1990). Another important pathway may be mediated by monoamine-oxidase in the extrahepatic tissues and in plasma by which chloracetaldehyde can be formed (Aeschlimann et al, 1996).

Methylene blue counteracts some of these metabolic pathways (Küpfer et al, 1996). It may act as an alternative electron acceptor, replacing the inhibited flavoproteins and thus restoring the mitochondrial respiratory chain (Harpey et al, 1986). It may also oxidate NADH, allowing dehydrogenation of the aldehydes (Hrushesky et al, 1985). It has also been found that MB may inhibit the plasma and extrahepatic monoamine oxidases (Aeschlimann et al, 1996). This may explain the preventive action of MB. To our knowledge, there are no known interactions with the pharmacokinetics of ifosfamide (Aeschlimann, 1998).

In our retrospective study, NCI-CTC neurotoxicity grade was lower during further ifosfamide treatment in the MB-treated group versus the untreated group of patients with ifosfamide-induced CNS effects. However, the numbers were small and the study was not randomized. Furthermore, because it was retrospective based on the patients' files, recovery times were only recorded in the daily progress notes and the shortest time recorded was 24 h with the exception of a few patients where multiple daily entries were made in the patient's medical file. The relatively high incidence of ifosfamide encephalopathy is probably due to the ifosfamide schedules used.

The literature mentions only six patients with ifosfamide-induced encephalopathy, which have been treated with MB, usually with spectacular results (Table 3). The first to present such a case was Küpfer et al (1994). Their patient recovered 30 min after administration of 50 mg of MB. They advised the prophylactic use of 3–4 × 50 mg $day^{-1}$ of MB. Later there were publications by Zulian et al (1995), Ferrero et al (1995) and Demandt and Wandt (1996). Alonso et al (1996) advised the same dose of MB that we used in our study, based on their observations after administration of 60 mg of MB. As we observed, even with the use of MB there may be a long recovery time. Koschuth et al (1996) presented a patient with a recovery of ifosfamide-induced encephalopathy 8 days after treatment with MB.

There are four studies on the duration of ifosfamide-induced encephalopathy in patients without a treatment with MB. They reported recovery times up to 29 days with an occasional fatal outcome. DiMaggio et al (1994) documented recovery times ranging from 2 to 7 days, Merimsky et al (1992) from 3 to 7 days and Curtin et al (1991) up to 13 days. Watkin et al (1989) even mentioned two patients in whom the encephalopathy lasted for more than 20 days.

There seems to be an advantage in using MB as a mean of shortening the duration of, or as a mean of preventing ifosfamide-induced encephalopathy, as was seen in our patients. Therefore, based on our experience and the available literature, we advise the intravenous use of MB in a dose of 6 × 50 mg $day^{-1}$ for treatment and 4 × 50 mg $day^{-1}$ either i.v. or orally for secondary prophylaxis of ifosfamide-induced encephalopathy.

### REFERENCES

Aeschlimann C, Cerny T and Küpfer A (1996) Inhibition of (mono) amine oxidase activity and prevention of ifosfamide encephalopathy by methylene blue. *Drug Metab Disp* **24**: 1336–1339

Aeschlimann C, Küpfer A, Schefer H and Cerny T (1998) Comparative pharmacokinetics of oral and intravenous ifosfamide/mesna/methylene blue therapy. *Drug Metab Disp* **26**: 883–890

Alonso JL, Nieto Y, López JA, Martín M and Díaz-Rubio E (1996) Ifosfamide encephalopathy and methylene blue: a case report. *Ann Oncol* **7:** 643–645

Cerny T, Castiglione M, Brunner K, Küpfer A, Martinelli G and Lind M (1990) Ifosfamide by continuous infusion to prevent encephalopathy. *Lancet* **335**: 175

Curtin JP, Koonings PP, Gutierrez M, Schlaerth JB and Morrow P (1991) Ifosfamide-induced neurotoxicity. *Gynecol Oncol* **42**: 193–196

Demandt M and Wandt H (1996) Erfolgreiche Behandlung von Ifosfamid-bedingten zentralnervösen Nebenwirkungen mit Methylenblau. *Dtsch Med Wochenschr* **121**: 575

DiMaggio JR, Brown R, Baile WF and Schapira D (1994) Hallucinations and ifosfamide-induced neurotoxicity. *Cancer* **73**: 1509–1514

Ferrero JM, Eftekari P, Largillier R, Dreyfus G and Namer M (1995) Traitement d'une encéphalopathie à l'ifosfamide par le bleu de méthylène. *Bull Cancer* **82**: 598–599

Harpey JP, Charpentier C and Coudé M (1986) Methylene blue for riboflavin-unresponsive glutarica ciduria type II. *Lancet* **I**: 391

Hrushesky WJ, Olshefski R, Wood P, Meshnick S and Eaton JW (1985) Modifying intracellular redox balance: an approach to improving therapeutic index. *Lancet* **I**: 565–567

Kaijser GP, Beijnen JH, Bult A and Underberg WJM (1994) Ifosfamide metabolism and pharmacokinetics. *Anticancer Research* **14**: 517–532

Koschuth A, Späth-Schwalbe E and Possinger K (1996) Methylenblau bei Ifosfamid-induzierter Enzephalopathie. *Dtsch Med Wochenschr* **121**: 1210

Küpfer A, Aeschlimann C, Wermuth B and Cerny T (1994) Prophylaxis and reversal of ifosfamide encephalopathy with methylene blue. *Lancet* **343**: 763–764

Küpfer A, Aeschlimann C and Cerny T (1996) Methylene blue and the neurotoxic mechanisms of ifosfamide encephalopathy. *Eur J Clin Pharmacol* **50**: 249–252

Kurowski V, Cerny T, Küpfer A and Wagner T (1991) Metabolism and pharmacokinetics of oral and intravenous ifosfamide. *J Cancer Res Clin Oncol* **117**: S148–S153

© 2000 Cancer Research Campaign 

Merimsky O, Reider-Groswasser I, Wigler N and Chaitchik S (1992) Encephalopathy in ifosfamide-treated patients. *Acta Neurol Scand* **86**: 521–525

Pratt OE, Rooprai HK, Shaw GK and Thomson AD (1990) The genesis of alcoholic brain tissue injury. *Alcohol Alcohol* **25:** 217–230

Van Tongelen K and Hawthorn J (1994) *A Practical Guide to EORTC Studies*. EORTC: Brussels

Watkin SW, Husband DJ, Green JA and Warenius HM (1989) Ifosfamide encephalopathy: a reappraisal. *Eur J Cancer Clin Oncol* **25**: 1303–1310

Zulian GB, Tullen E and Maton B (1995) Methylene blue for ifosfamide associated encephalopathy. *N Engl J Med* **332**:1239–1240

 *© 2000 Cancer Research Campaign*
